# Geometry-Dependent Photonic Nanojet Formation and Arrays Coupling

**DOI:** 10.3390/nano16020136

**Published:** 2026-01-20

**Authors:** Zehua Sun, Shaobo Ge, Lujun Shen, Junyan Li, Shibo Xu, Jin Zhang, Yingxue Xi, Weiguo Liu

**Affiliations:** Shaanxi Province Key Laboratory of Thin Films Technology and Optical Test, School of Optoelectronic Engineering, Institute for Interdisciplinary and Innovation Research, Xi’an Technological University, Xi’an 710021, China; sunzehua@st.xatu.edu.cn (Z.S.); shenlujun@st.xatu.edu.cn (L.S.); lijunyan@st.xatu.edu.cn (J.L.); xushibo@st.xatu.edu.cn (S.X.); j.zhang@xatu.edu.cn (J.Z.); xiyingxue@xatu.edu.cn (Y.X.); wgliu@163.com (W.L.)

**Keywords:** photonic nanojet, array coupling, photonic hooks, geometric configuration

## Abstract

This work systematically investigates photonic nanojet (PNJ) planar arrays formed by periodic arrangements of dielectric microstructures with four geometric configurations: cylinders, cones, truncated pyramids, and pyramids, focusing on the effects of geometry, array arrangement, and array sparsity on PNJ formation and coupling behavior. Full-wave finite-difference time-domain simulations were performed to analyze optical field distributions under different array conditions. The results indicate that under approximately infinite array conditions, different geometries exhibit markedly different coupling responses. Cylindrical and truncated pyramid structures are more susceptible to inter-element scattering, leading to pronounced multistage focusing, whereas pyramid and cone structures maintain higher spatial stability due to dominant localized tip-focusing mechanisms. For the central elements, the maximum PNJ intensity is about 16.4 a.u. for cylindrical structures and 33.5 a.u. for truncated pyramid structures, while significantly higher intensities of approximately 47.5 a.u. and 93 a.u. are achieved for pyramid and cone structures, respectively. In contrast, the FWHM remains nearly constant for all geometries under different array conditions, indicating that lateral focusing is primarily governed by geometry rather than array arrangement. By tuning the array spacing, the inter-element coupling strength can be continuously weakened, and different geometries require distinct sparsity levels to reach the weak-coupling limit. These results establish the dominant role of geometric configuration in PNJ planar arrays and provide guidance for their predictable design.

## 1. Introduction

The photonic nanojet (PNJ) is a high-intensity, subwavelength-localized light beam formed on the exit side of a dielectric microstructure, whose lateral dimension can surpass the diffraction limit while maintaining an axial extension over multiple wavelengths [1,2,3]. Owing to its ability to achieve significant optical field enhancement without relying on plasmonic resonance [4], the PNJ exhibits broad application prospects in super-resolution imaging [5,6], optical manipulation [7], enhanced light–matter interaction [8], and nanofabrication [9,10]. In recent years, as the demand for parallelization and large-area optical field construction in related applications has continuously increased [11], periodically arranged planar PNJ arrays have emerged as an important platform for scalable and controllable optical field engineering [12].

In existing studies, PNJ planar arrays are primarily realized using dielectric microsphere arrays [13]. Regularly arranged microsphere arrays can generate periodic distributions of PNJs on the exit side, thereby enabling multipoint parallel focusing and enhanced optical field control [14]. Such structures possess certain advantages in experimental implementation and theoretical modeling and are therefore widely applied in imaging, optical sensing, and surface-enhanced studies [15]. However, constrained by the highly symmetric geometry of microspheres, the resulting PNJs typically exhibit limited propagation lengths and relatively fixed spatial profiles, which to some extent restrict the application potential of PNJ arrays in long working-distance or more complex optical field modulation scenarios [16,17].

In contrast, nonspherical dielectric microstructures have been demonstrated to generate PNJs with richer spatial characteristics under single-element conditions [18,19,20,21], such as extended propagation distances, flexible control of focal positions, and the introduction of asymmetric optical field distributions [22,23,24]. Nevertheless, existing studies have predominantly focused on individual structures or isolated device levels [25,26]. Systematic investigations that directly compare different nonspherical geometries under identical periodic array conditions remain limited. Because nonspherical dielectric structures usually possess anisotropic geometric features and more complex refractive pathways, their collective optical response in periodic arrays cannot be straightforwardly inferred from single-particle behavior [27,28]. The optical field interactions between adjacent elements and their dependence on geometric configuration remain insufficiently understood [29,30,31], which to some extent limits the further development of nonspherical PNJ arrays from structural design to engineering applications.

Therefore, this work systematically investigates the optical behavior of PNJ planar arrays composed of four representative geometric configurations—cylinders, cones, truncated pyramids, and pyramids—under periodic arrangement conditions. The cylindrical and conical structures, respectively, represent axisymmetric focusing scenarios dominated by planar interfaces and continuously inclined interfaces [32,33,34,35], while the truncated pyramid and pyramid structures introduce pronounced geometric truncation characteristics through finite top facets or sharp tips [20,36]. These configurations are used to characterize PNJ formation behaviors under conditions of finite cone angles and localized tip focusing. The four geometric configurations collectively encompass typical geometric features ranging from planar refraction and continuous inclined refraction to tip-induced localized focusing. By performing a unified, geometry-spanning comparison within periodic arrays, this study reveals how array-induced coupling reshapes PNJ morphology in a geometry-dependent manner, beyond what can be predicted from single-element analyses. The results provide new physical insight into the interplay between particle geometry and collective coupling effects, offering a systematic basis for the predictable design and application expansion of nonspherical PNJ planar arrays.

## 2. Materials and Methods

**Figure 1:**
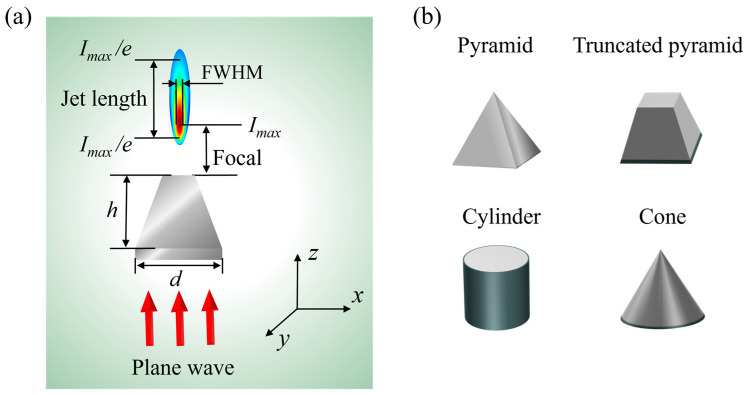
Schematic illustrations of the structural models and optical field distributions: (**a**) the main parameters of the PNJ; (**b**) different geometric configurations.

In this study, numerical simulations of PNJs generated by microstructures with different geometric configurations and array arrangements were performed using the full-wave finite-difference time-domain (FDTD) method based on vector electromagnetic wave theory. Figure 1a presents a schematic illustration of PNJ formation. The incident light source was set as a plane wave with a wavelength of 632.8 nm, normally incident along the normal direction of the microstructure base. The *z*-axis is defined as the light propagation direction, while the *x*–*y* plane represents the transverse plane. To ensure numerical accuracy and computational efficiency, the spatial grid step was set to be smaller than one-sixth of the incident wavelength. When simulating the response of periodic arrays, periodic boundary conditions (PBCs) were applied at the transverse boundaries of the computational domain, such that the modeled array represents an effectively infinite periodic array, hereafter denoted as array (∞). For simulations of finite arrays and single microstructures, perfectly matched layers (PMLs) were employed at the transverse boundaries to effectively absorb outgoing electromagnetic waves and eliminate nonphysical reflections.

The geometric and optical characteristics of PNJs are quantitatively characterized by four parameters: focal length, maximum intensity *I*_max_, full width at half maximum (FWHM), and jet length, as illustrated in Figure 1a. The focal length is defined as the axial distance from the exit tip of the microstructure to the position of the *I*_max_ along the optical axis. The transverse beam width is described by the FWHM measured along the *x*-direction at the focal plane, which is used to evaluate the degree of lateral localization of the jet. The jet length is defined as the axial distance between two positions along the optical axis where the intensity decays from its peak value to *I*_max_/e, with e denoting the base of natural logarithms [37,38,39]. Figure 1b shows the four representative geometric configurations investigated in this work, namely pyramid, truncated pyramid, cylinder, and cone structures. All structures have a base width of 7 μm and a uniform height of 2.4 μm, while the top surface width of the truncated pyramid structure is 1.2 μm. It should be noted that the microstructures in our simulations are deposited on a substrate, as depicted in Figure 1a. The substrate material and the microstructures are both assigned identical parameters (PMMA, λ = 632.8 nm, n = 1.48) to eliminate additional effects introduced by material interfaces.

## 3. Results and Discussion

### 3.1. Differences in Array Coupling of PNJs for Different Geometric Configurations

**Figure 2:**
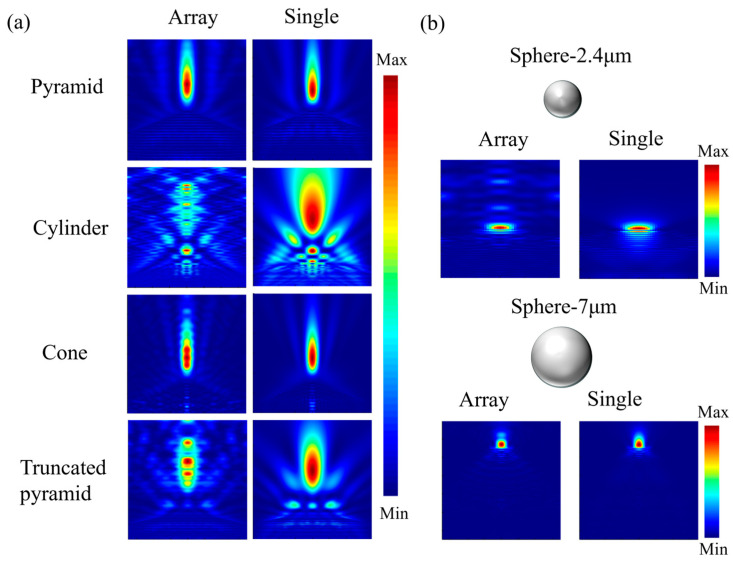
Optical field distributions of different PNJ-generating configurations under array conditions (left column) and for single elements (right column). (**a**) pyramid, cylinder, cone, truncated pyramid; (**b**) microsphere.

Figure 2 comparatively illustrates the PNJ morphologies generated by five geometric configurations—microsphere, pyramid, cylinder, cone, and truncated pyramid—under both single-element and array conditions. The array condition corresponds to an effectively infinite periodic array implemented via transverse periodic boundary conditions, denoted as array (∞). A key observation is that the influence of array coupling on PNJ formation strongly depends on the underlying geometry, with the microsphere structure exhibiting the highest robustness against array-induced modulation.

For the microsphere structure, two reference cases are considered in Figure 2b, with diameters of 2.4 μm and 7 μm, which are deliberately chosen to match the height (2.4 μm) and base width (7 μm) of the nonspherical microstructures, respectively. Under the single-element condition, both microspheres generate a classical, axially symmetric, and well-confined PNJ, consistent with previously reported microsphere-based PNJ characteristics. Notably, after introducing the array arrangement, the PNJ characteristics of both microsphere cases remain largely unchanged. The jets preserve their axial symmetry, focal positions, and transverse localization, with only weak periodic intensity modulation along the propagation direction resulting from coherent superposition between neighboring spheres. These weak oscillations do not significantly alter the overall PNJ morphology, demonstrating that microsphere-based PNJs are predominantly governed by intrinsic volume-focusing mechanisms and are only weakly affected by array coupling. This behavior establishes the microsphere as a robust and geometry-independent reference baseline for evaluating array-induced PNJ modulation in nonspherical structures.

For the pyramid structure in Figure 2a, under the single-element condition, its tip geometry produces a highly localized PNJ with a well-defined axial profile. Under array conditions, this PNJ morphology remains essentially unchanged, with only weak background interference features observed. The stability of the jet axis, peak position, and spatial profile indicates that localized tip-dominated focusing suppresses array coupling effects, although the robustness is still slightly weaker than that observed for the microsphere reference.

By contrast, the cylindrical, conical, and truncated pyramid structures all exhibit pronounced PNJ reconstruction under array conditions. For the cylindrical structure, the PNJ generated under the single-element condition is relatively smooth, whereas in the array it is strongly influenced by lateral coupling effects: the continuous jet is modulated into multiple intensity maxima distributed along the propagation direction, its transverse localization remains relatively well-confined.

The conical structure is capable of generating a slender and continuous PNJ under the single-element condition. Although its overall axial continuity is preserved in the array, distinct periodic intensity oscillations emerge along the propagation direction, indicating a higher sensitivity to axial superposition effects compared with the microsphere baseline.

The truncated pyramid structure exhibits the most pronounced array-induced modulation behavior. The PNJ that is localized near the flat exit surface under the single-element condition is reconstructed into multiple discrete refocusing regions within the array. Compared with the microsphere reference cases in Figure 2b, this structure shows significantly enhanced array-induced complexity, highlighting the critical role of geometric anisotropy and exit-surface topology in PNJ modulation.

**Figure 3:**
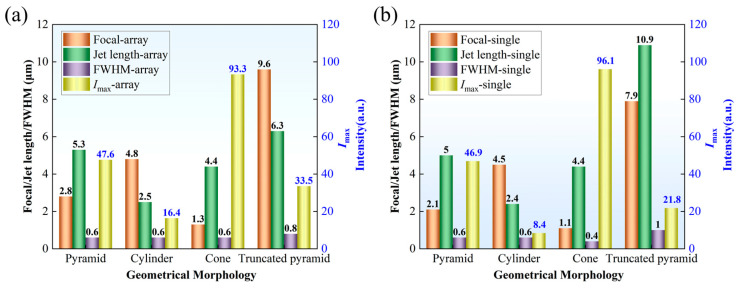
Main parameters of PNJ for different geometric configurations: (**a**) array (∞); (**b**) single.

Figure 3 provides a quantitative summary of the above morphological differences. The results show that, for the pyramid structure, the jet length, transverse full width at half maximum, and peak intensity in the array remain essentially consistent with those under the single-element condition, further confirming its weak sensitivity to array coupling from a parametric perspective. In contrast, for the cylindrical structure, the transverse FWHM under array conditions remains comparable to that of the single-element case, while both the peak intensity and the jet length increase. This behavior suggests that array coupling in the cylindrical configuration predominantly enhances axial field buildup through constructive superposition, without inducing noticeable transverse broadening of the photonic nanojet. For the conical structure, the jet length, peak intensity, and transverse size under array conditions remain largely comparable to those of the single-element case, indicating a weak influence of array coupling on its photonic nanojet characteristics. The truncated pyramid structure shows the most complex variation in PNJ parameters, with its jet peak position and intensity undergoing significant changes depending on the array condition, demonstrating a high sensitivity to geometric arrangement and interactions with neighboring structures. These results indicate that geometric configuration not only determines the fundamental PNJ morphology under single-element conditions but also plays a dominant role in governing the coupling mechanisms and energy distribution characteristics under array configurations.

### 3.2. PNJs Generated by Different Geometric Structures Under Finite Array Conditions

#### 3.2.1. Cylinder

**Figure 4:**
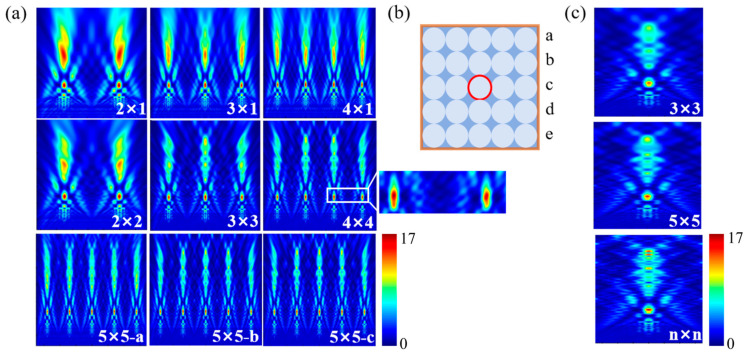
PNJ distributions of the cylindrical geometry under finite array arrangements: (**a**) optical field distributions of cylindrical geometry with different array layouts; (**b**) Schematic diagram of a 5×5 array. a, b, c, d, e respectively represent the 1st, 2nd, 3rd, 4th, and 5th rows of the array. The red circle represents the central unit of the array; (**c**) PNJ of the central element for different cylindrical array configurations.

Extending the analysis from approximately infinite arrays to finite array arrangements, Figure 4a illustrates the modulation behavior of PNJs generated by cylindrical structures with different array sizes and configurations. The white numerical labels denote different array layouts, whose geometric schematics are shown in Figure 4b. For the 5 × 5 array layout, 5 × 5—a, 5 × 5—b, and 5 × 5—c correspond to the PNJ light field patterns from the first, second, and third rows of the array, respectively, as shown in Figure 4b. Unlike the single-element or approximately infinite array cases, the PNJs produced by cylindrical structures under finite array conditions exhibit more complex multistage focusing characteristics.

Specifically, cylindrical structures typically form two primary focal regions along the axial direction: a first-order focus located near the rear surface of the structure and a second-order focus positioned at a farther axial distance. In small-scale arrays (such as 2 × 1 and 3 × 1), a well-defined first-order focus is already formed, with its position remaining largely consistent with that under the single-element condition. In the 4 × 4 array configuration, the first-order focus can be clearly observed to appear simultaneously behind multiple adjacent cylinders (as highlighted and magnified in the inset to the right of Figure 4a, with minimal variation in spatial position and morphology, indicating that this first-order focus possesses strong geometric stability and is insensitive to the array scale.

Unlike the first-order focus, the second-order focus generated by cylindrical structures in finite arrays exhibits a pronounced dependence on the array size. As shown in the figure, with increasing array size, the overall intensity of the second-order focus gradually decreases and its axial contrast is significantly reduced, indicating that the superposition of scattered fields from neighboring cylinders suppresses the formation of the second-order focus within the interior region of the array. Moreover, the spatial morphology of the second-order focus shows clear position-dependent differences within the array. For cylindrical elements located in the central region of the array, the second-order focus maintains a stable axisymmetric profile without noticeable bending. In contrast, for cylinders positioned at the outermost edges of the array, the second-order focus exhibits varying degrees of deflection in both small- and large-scale arrays, forming curved PNJs, namely photonic hook (PH) structures [40,41,42]. This phenomenon is consistent with the theoretical analysis by Yang et al. [41], which attributes curved PNJ formation to asymmetric dielectric boundaries and nonuniform phase accumulation, and its physical origin lies in the asymmetric refraction and scattering pathways experienced by cylindrical structures at the edges of finite arrays. These results indicate that, for cylindrical geometries, the array size systematically influences the multistage focusing behavior of PNJs by modulating the boundary conditions, such that the first-order focus remains stable during scale expansion, whereas the intensity and morphology of the second-order focus exhibit much higher sensitivity to the array size and element position.

To further clarify the influence of boundary effects, Figure 4c presents the PNJ distributions corresponding to the central cylindrical element in 3 × 3, 5 × 5, and n × n arrays (marked by the red position in Figure 4b), where n × n denotes a sufficiently large two-dimensional periodic array approaching the infinite-array limit (n → ∞), corresponding to the array (∞) configuration defined earlier. It can be observed that, as the array size increases, the PNJ distribution of the central element gradually converges toward that of an approximately infinite array. Correspondingly, Table 1 provides a systematic quantitative summary of the PNJ characteristics generated by the central element under different array configurations shown in Figure 4c. As the array size increases from 3 × 3 to 5 × 5 and further to n × n, the focal length of the PNJ remains essentially constant at approximately 4.75–4.8 μm, indicating that array boundaries have a negligible influence on the axial focal position. Meanwhile, the jet length gradually decreases from 2.94 μm with increasing array size and eventually stabilizes, reaching its minimum value in the n × n array, suggesting that the reduction in boundary effects facilitates a more compact axial energy distribution. In addition, the PNJ FWHM remains at 0.56 μm for all array configurations, demonstrating that its lateral focusing capability is insensitive to variations in array size.

#### 3.2.2. Ftrustum of a Pyramid

**Figure 5:**
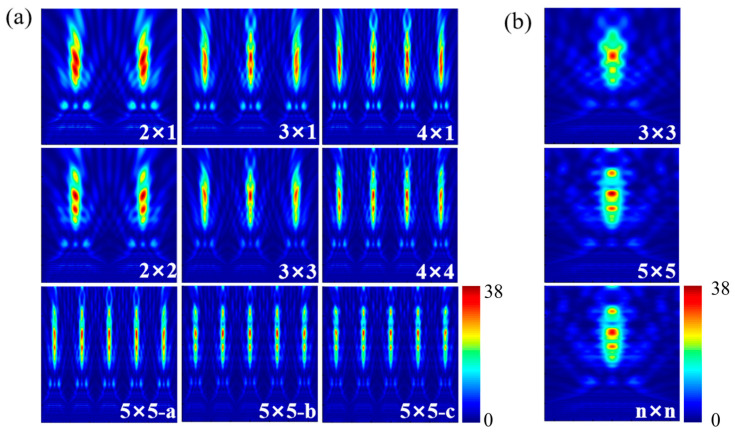
PNJ distributions of the truncated pyramid geometry under finite array arrangements: (**a**) optical field distributions of the truncated pyramid geometry under different array layouts; (**b**) PNJ of the central element of the truncated pyramid geometry for different array configurations.

The evolution behavior of PNJs generated by truncated pyramid structures under finite array arrangements is shown in Figure 5. Compared with cylindrical structures, truncated pyramids exhibit much stronger structural sensitivity of their PNJs under finite array conditions. As shown in Figure 5a, in small-scale arrays (such as 2 × 1 and 3 × 1), the PNJs of truncated pyramid structures display pronounced axial segmentation characteristics, indicating that their focusing processes are jointly modulated by sidewall refraction and refocusing at the planar exit interface. As the array expands from one-dimensional to two-dimensional configurations, particularly in 3 × 3 and 4 × 4 arrays, the PNJs of truncated pyramid structures gradually evolve into multiple discrete high-intensity focal regions along the propagation direction, exhibiting typical multistage refocusing behavior. Notably, similar to the case of cylindrical structures, deflection of the second-order and subsequent focal regions can also be observed at the outermost elements of the array, leading to the formation of curved PNJs [41,42,43], whereas truncated pyramids located in the central region of the array maintain an approximately axisymmetric PNJ profile without noticeable bending. However, unlike the relatively stable first-order focus observed in cylindrical structures, the focal positions of all focusing stages in truncated pyramid structures vary significantly with array size, and both the axial spacing and relative intensity of these focal regions are strongly influenced by the superposition of scattered fields from neighboring elements. This indicates that, in finite arrays, the PNJs of truncated pyramid structures are no longer governed by a single interface but are instead jointly determined by progressive focusing induced by finite cone-angle sidewalls and refocusing effects introduced by the flat top interface, thereby amplifying the modulation effect of array arrangement on PNJ morphology.

As shown in Figure 5b, with further increases in array size, the axial distribution of the multistage focusing structure gradually becomes more regular, while the segmented characteristics are still preserved and do not converge to the continuous jet morphology observed in cylindrical or conical structures. According to Table 2, in 3 × 3, 5 × 5, and n × n arrays, the focal length of the truncated pyramid PNJ remains within the range of approximately 9.3–9.8 μm with relatively small variation, whereas the jet length exhibits a clear decreasing trend with increasing array size, indicating that the axial distribution of multistage focusing becomes progressively more compact. Meanwhile, the FWHM remains constant at 0.84 μm under all array conditions, demonstrating that the lateral focusing capability of the truncated pyramid structure is insensitive to array size. By contrast, the *I*_max_ reaches its highest value in the 5 × 5 array and then slightly decreases in the n × n array, suggesting that truncated pyramid structures can achieve significant field enhancement through inter-element coupling in finite-sized arrays, while their multistage refocusing characteristics limit the continuous accumulation of energy within a single focal region as the array approaches the infinite limit.

#### 3.2.3. Pyramid Geometry and Cone

**Figure 6:**
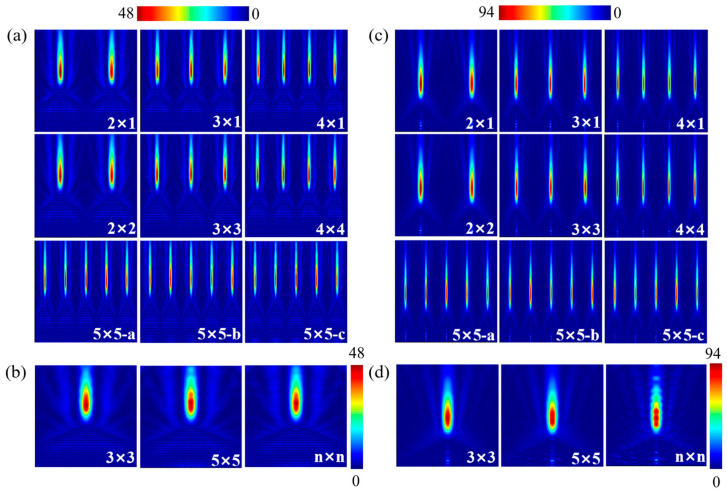
PNJ distributions of the pyramidal and conical geometries under finite array arrangements: (**a**) optical field distributions of the pyramidal geometry under different array layouts; (**b**) PNJ optical field distributions of the central element of the pyramidal geometry for different array configurations; (**c**) optical field distributions of the conical geometry under different array layouts; (**d**) PNJ optical field distributions of the central element of the conical geometry for different array configurations.

A further comparison of the PNJ distributions generated by pyramid and cone structures under finite array arrangements is shown in Figure 6. As can be seen from Figure 6a,c, neither the pyramid nor the cone structures exhibit pronounced morphological reconstruction of their PNJs under different array sizes and configurations; the principal axis direction, overall continuity, and localization characteristics of the jets remain essentially unchanged, indicating that these two tip-based structures are significantly less sensitive to array arrangement than the cylindrical and truncated pyramid structures. In contrast, the influence of the array is mainly reflected in subtle variations in the central elements, as shown in Figure 6b,d. The PNJs of the central pyramid elements almost completely overlap across different array sizes [44], while the central cone elements also maintain good continuity, although their axial intensity distribution and jet length show slight variations with array size, reflecting weak axial superposition modulation in cone arrays. Correspondingly, Table 3 and Table 4 summarize the main PNJ parameters of the central elements for pyramid and cone structures, respectively, under different array sizes. As shown in Table 3, the focal length, jet length, and *I*_max_ of the central pyramid PNJ vary only slightly among the 3 × 3, 5 × 5, and n × n arrays, with the focal length maintained at approximately 2.5–2.9 μm, the jet length stabilized at about 4.7–5.3 μm, the FWHM consistently fixed at 0.62 μm, and *I*_max_ exhibiting no significant dependence on array size, indicating highly stable intrinsic focusing characteristics of pyramid PNJs under finite array conditions. By comparison, Table 4 shows that the focal length and jet length of the central cone PNJ exhibit mild fluctuations with array size, but the overall variations remain limited, with focal lengths distributed in the range of 0.95–1.3 μm and jet lengths maintained at approximately 4.35–4.64 μm, while the FWHM remains constant at 0.55 μm. Meanwhile, although the *I*_max_ of the cone structure shows some variation under different array conditions, it remains at a relatively high level overall, indicating that its focusing efficiency exhibits only weak dependence on array arrangement.

From a physical mechanism perspective, although both pyramid and cone structures possess tip geometries, the formation pathways of their PNJs are fundamentally different. In pyramid structures, the PNJ is primarily governed by strong localized convergence induced by multiphase refraction near the tip, with the focusing process completed over a very short axial distance, thereby significantly reducing the jet’s dependence on external phase perturbations and scattered fields from neighboring elements. In contrast, the PNJ of cone structures relies on a progressive refraction and energy accumulation process along the axial direction, resulting in a more spatially extended effective focusing region, which makes it more susceptible to modulation by inter-element coupling and axial superposition effects within the array.

### 3.3. Regulation of PNJ Coupling by Array Sparsity

The foregoing results indicate that different geometric configurations exhibit markedly different coupling sensitivities under array conditions. We therefore consider whether, under periodic arrangements, the effective coupling strength between adjacent structures can be continuously tuned by adjusting the array sparsity, such that the PNJ behavior gradually converges toward the single-element limit. To this end, the array spacing is introduced as an additional degree of freedom to investigate this issue.

**Figure 7:**
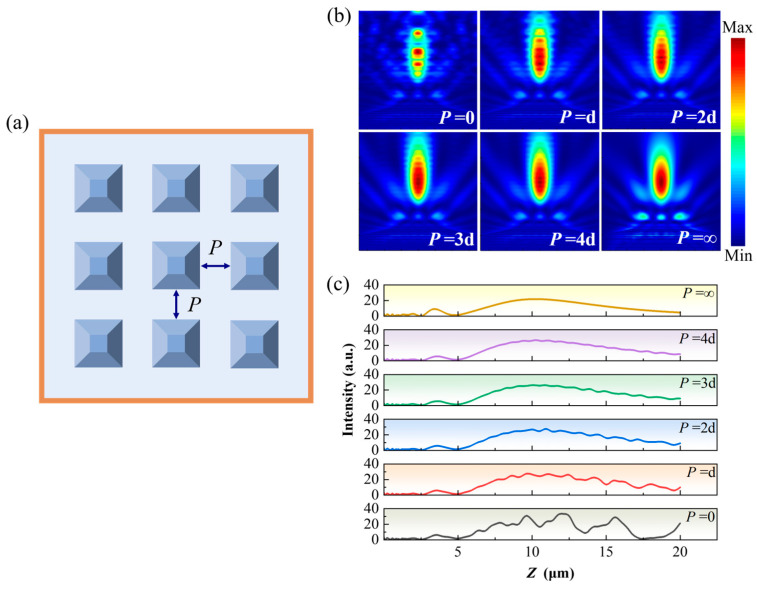
Effect of array spacing on PNJ field distributions of the truncated pyramid geometry: (**a**) schematic of the array arrangement; (**b**) PNJ field distributions under different array spacings; (**c**) PNJ optical field propagation along the *z* direction for different array spacings.

Figure 7 illustrates the PNJ distributions and propagation characteristics generated by truncated pyramid structures under different array spacing conditions. Specifically, Figure 7a presents a schematic of the array arrangement for the truncated pyramid structures, where *P* denotes the center-to-center spacing between adjacent elements. The limiting case *P* = 0 corresponds to a zero-gap configuration with neighboring elements in direct contact, whereas *P* = ∞ denotes the single-element condition with infinitely separated elements; Figure 7b compares the spatial distributions of PNJs at different array spacings; and Figure 7c shows the corresponding optical field propagation profiles along the *z* direction. When the array spacing is small, the scattered fields between neighboring truncated pyramid structures undergo strong superposition, causing the PNJs to exhibit pronounced multistage refocusing behavior along the propagation direction, with significant axial intensity oscillations, reflecting optical field evolution dominated by strong array coupling. As the array spacing is gradually increased, the axial modulation of the jet is progressively weakened, background interference structures are effectively suppressed, and the PNJ morphology becomes smoother. It can be further observed that when the array spacing increases to a certain extent, although the structures remain periodically arranged, the PNJ generated by the central element becomes highly consistent in both morphology and propagation characteristics with those of a single-element structure, as shown by the comparison between large *P* values and *P* = 0 in Figure 7b,c. This demonstrates that increasing array sparsity can progressively reduce the effective coupling between adjacent structures in periodic arrays, driving the PNJ behavior toward the noninteracting single-element limit.

**Figure 8:**
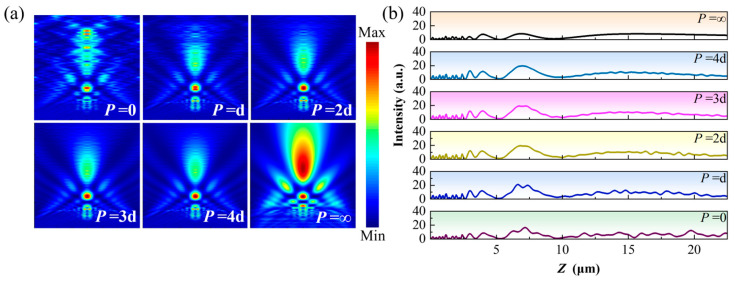
Effect of array spacing on PNJ field distributions of the cylindrical geometry: (**a**) PNJ field distributions under different array spacings; (**b**) PNJ optical field propagation along the *z* direction for different array spacings.

Under the same array-spacing modulation conditions, the PNJs generated by cylindrical structures exhibit response characteristics to array sparsity that are markedly different from those of truncated pyramid structures, as shown in Figure 8. When the array spacing is small, strong lateral coupling between adjacent cylinders persists, and the PNJs display pronounced multistage axial intensity oscillations, with clearly discernible second-order focal regions and strong background interference, indicating that array coupling remains dominant in the optical field formation process. As the array spacing gradually increases, the PNJs generated by cylindrical structures begin to evolve noticeably. At *P* = 2d and *P* = 3d (where d denotes the width of the microstructure, as defined in Figure 1a), the amplitude of axial modulation induced by array coupling is partially reduced. When the array spacing is further increased to *P* = 4d, the overall PNJ morphology becomes smoother, and its axial intensity distribution more closely approaches that of *P* = 0; however, compared with the single configuration, discernible signatures of array-induced modulation are still retained. This trend is further confirmed by the optical field propagation profiles along the *z* direction shown in Figure 8b: as the array spacing increases, axial intensity oscillations are progressively suppressed, but the decay process is relatively slow, requiring larger array spacings for the PNJ of cylindrical structures to approach the single-element limit. These results indicate that, compared with truncated pyramid structures, cylindrical structures in periodic arrays are more sensitive to lateral coupling with neighboring elements, and their PNJ formation involves a longer effective focusing region, thereby necessitating higher array sparsity to effectively weaken array coupling effects.

**Figure 9:**
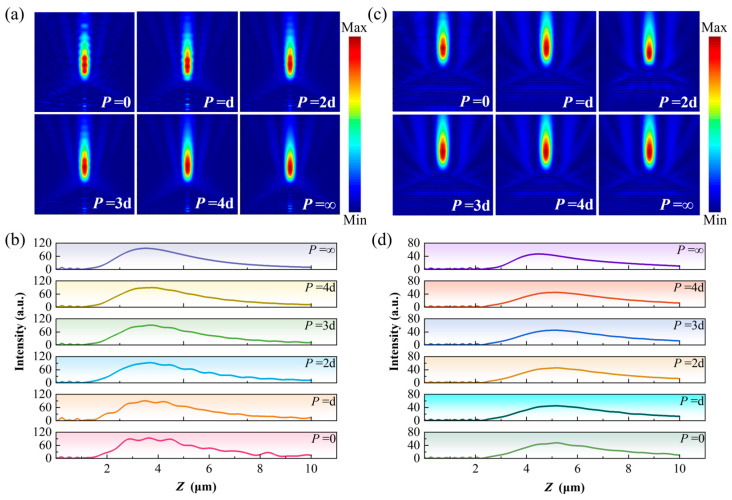
Effect of array spacing on PNJ field distributions of the conical and pyramidal geometries: (**a**) PNJ field distributions of the conical geometry under different array spacings; (**b**) PNJ optical field propagation along the *z* direction for the conical geometry under different array spacings; (**c**) PNJ field distributions of the pyramidal geometry under different array spacings; (**d**) PNJ optical field propagation along the *z* direction for the pyramidal geometry under different array spacings.

Under the same array-spacing modulation conditions, the responses of PNJs generated by cone and pyramid structures to array sparsity are further examined, as shown in Figure 9. Figure 9a,b present the PNJ distributions of cone structures under different array spacings, indicating that the influence of array sparsity on cone-generated PNJs is relatively limited. Nevertheless, from the corresponding optical field propagation curves along the *z* direction, it can still be observed that, as the array spacing decreases, the axial intensity distribution of the jet exhibits a certain degree of oscillatory modulation, suggesting that weak axial superposition effects between adjacent structures persist. As the array spacing increases, this modulation gradually diminishes, and the PNJ characteristics progressively approach those of the single-element structure.

By contrast, pyramid structures exhibit greater robustness with respect to variations in array spacing, as shown in Figure 9c,d. Under different array spacing conditions, the PNJs generated by pyramid structures remain nearly identical in spatial morphology, with no pronounced changes in focal position, jet length, or degree of lateral localization as *P* varies. The corresponding optical field propagation curves along the *z* direction further demonstrate that the axial intensity distributions of pyramid structures are highly overlapped across different array spacings, indicating that array sparsification has a negligible influence on their PNJ behavior.

In summary, the coupling effects of PNJs in periodic arrays can be continuously regulated by adjusting the array sparsity. As the array spacing increases, the optical field interactions between adjacent elements are progressively weakened, and the spatial distribution and axial propagation characteristics of PNJs gradually converge toward the limiting behavior of single-element structures. However, the array sparsity required to reach this weak-coupling limit differs significantly among different geometric configurations: structures exhibiting stronger intrinsic coupling and larger spatial scales of the focusing process require larger array spacings to effectively suppress the influence of neighboring elements, whereas structures with weaker array coupling can maintain stable PNJ behavior even at relatively small spacings. These results demonstrate that, by appropriately selecting the geometric configuration and array sparsity, effective control over array coupling strength can be achieved while preserving periodic arrangements.

## 4. Conclusions

This study clarifies the fundamental role of geometric configuration in governing PNJ coupling behaviors within periodic arrays. By systematically comparing different dielectric geometries, it is shown that the intrinsic focusing mechanisms of individual structures largely determine their tolerance to array-induced interactions, leading to distinct coupling regimes across geometries. Structures characterized by extended focusing processes exhibit stronger sensitivity to neighboring elements, whereas tip-dominated geometries inherently suppress array coupling and preserve stable PNJ characteristics. Importantly, array sparsity is identified as an effective control parameter for tuning inter-element interactions, enabling a continuous transition from collective array behavior to single-element-like responses. The required sparsity to achieve weak coupling is found to be geometry-dependent, reflecting the interplay between focusing length scale and spatial field overlap. These insights establish practical design guidelines for PNJ planar arrays and support their scalable implementation in parallel optical manipulation, subwavelength imaging, and nanofabrication systems.

## Figures and Tables

**Table 1:** Main PNJ parameters of the central element for the cylindrical geometry under different array arrangements.

Array Numbers	Focal/(μm)	Jet Length/(μm)	FWHM/(μm)	*I*_max_/(a.u.)
3 × 3	4.75	2.94	0.56	13.86
5 × 5	4.8	2.63	0.56	15.19
n × n	4.75	2.5	0.56	16.42

**Table 2:** Main PNJ parameters of the central element for the truncated pyramid geometry under different array arrangements.

Array Numbers	Focal/(μm)	Jet Length/(μm)	FWHM/(μm)	*I*_max_/(a.u.)
3 × 3	9.33	9.5	0.84	33.22
5 × 5	9.8	5.96	0.84	37.87
n × n	9.6	6.3	0.84	33.46

**Table 3:** Main PNJ parameters of the central element for the pyramidal geometry under different array arrangements.

Array Numbers	Focal/(μm)	Jet Length/(μm)	FWHM/(μm)	*I*_max_/(a.u.)
3 × 3	2.5	4.74	0.62	47.72
5 × 5	2.9	5.2	0.62	47.12
n × n	2.75	5.3	0.62	47.56

**Table 4:** Main PNJ parameters of the central element for the conical geometry under different array arrangements.

Array Numbers	Focal/(μm)	Jet Length/(μm)	FWHM/(μm)	*I*_max_/(a.u.)
3 × 3	1.3	4.45	0.55	92.43
5 × 5	0.95	4.64	0.55	90.212
n × n	1.25	4.35	0.55	93.2883

## Data Availability

The authors confirm that the data supporting the findings of this study are available within the article.

## Abbreviations

The following abbreviations are used in this manuscript:

PNJ	Photonic nanojet
*I*_max_	Maximum light intensity
